# Transcriptome-Wide Identification and Expression Analysis of the NAC Gene Family in Tea Plant [*Camellia sinensis* (L.) O. Kuntze]

**DOI:** 10.1371/journal.pone.0166727

**Published:** 2016-11-17

**Authors:** Yong-Xin Wang, Zhi-Wei Liu, Zhi-Jun Wu, Hui Li, Jing Zhuang

**Affiliations:** Tea Science Research Institute, College of Horticulture, Nanjing Agricultural University, Nanjing 210095, China; National Bureau of Plant Genetic Resources, INDIA

## Abstract

In plants, the NAC (NAM-ATAF1/2-CUC) family of proteins constitutes several transcription factors and plays vital roles in diverse biological processes, such as growth, development, and adaption to adverse factors. Tea, as a non-alcoholic drink, is known for its bioactive ingredients and health efficacy. Currently, knowledge about NAC gene family in tea plant remains very limited. In this study, a total of 45 *CsNAC* genes encoding NAC proteins including three membrane-bound members were identified in tea plant through transcriptome analysis. CsNAC factors and Arabidopsis counterparts were clustered into 17 subgroups after phylogenetic analysis. Conserved motif analysis revealed that CsNAC proteins with a close evolutionary relationship possessed uniform or similar motif compositions. The distribution of NAC family MTFs (membrane-associated transcription factors) among higher plants of whose genome-wide has been completed revealed that the existence of doubled TMs (transmembrane motifs) may be specific to fabids. Transcriptome analysis exhibited the expression profiles of *CsNAC* genes in different tea plant cultivars under non-stress conditions. Nine *CsNAC* genes, including the predicted stress-related and membrane-bound genes, were examined through qRT-PCR (quantitative real time polymerase chain reaction) in two tea plant cultivars, namely, ‘Huangjinya’ and ‘Yingshuang’. The expression patterns of these genes were investigated in different tissues (root, stem, mature leaf, young leaf and bud) and under diverse environmental stresses (drought, salt, heat, cold and abscisic acid). Several *CsNAC* genes, including *CsNAC17* and *CsNAC30* that are highly orthologous to known stress-responsive *ANAC072/RD26* were identified as highly responsive to abiotic stress. This study provides a global survey of tea plant NAC proteins, and would be helpful for the improvement of stress resistance in tea plant *via* genetic engineering.

## Introduction

Tea is a non-alcoholic drink known for its bioactive ingredients and health efficacy. Tea plant [*Camellia sinensis* (L.) O. Kuntze], which originated from the southwest region of China, had been cultivated and utilized for thousands of years, and is planted worldwide nowadays [1]. Surviving in the wild, tea plant often suffers from numerous detrimental environmental factors, such as extreme temperature, drought and salinity [2, 3]. Enhanced environmental receptivity may significantly help improve the yield and quality of tea plant. The potential adversity resistance of plants is often determined by expressing stress-inducible genes that are regulated by specific transcription factors (TFs) [4]. TFs widely exiting in plants regulate the expression of target genes by binding directly or indirectly with specific *cis*-regulatory elements.

NAC (NAM-ATAF1/2-CUC) TF is a large TF family widely existing in plants. Most members of the NAC family contain a conserved NAC domain (~150 aa), which consists of five subdomains (A, B, C, D, and E), in the N-terminus. In general, subdomains A, C, and D are tightly conserved, whereas subdomains B and E are divergent [5]. Subdomains B and E may be closely related to the functional diversity of NAC genes, subdomain A may participate in dimer formation, and subdomains C and D are mainly involved in DNA binding (DB) [6–8]. The C-terminal regions of NAC TFs are highly variable, which might function as transcriptional activators or repressors that regulate the expression of downstream genes [9]. Moreover, some NAC TFs contain transmembrane motifs (TMs), which are working for the plasma membrane or endoplasmic anchoring, at the C-terminal end [10]. X-ray crystallography of Arabidopsis ANAC019 and rice SNAC1 revealed that the NAC domain monomer exhibits a novel TF folding pattern, consisting of a twisted antiparallel β-sheet, which is devoted to DB and encircled by an α-helix element on both sides [6, 7].

Recent genome-wide analyses have identified 117 NAC genes in Arabidopsis (*Arabidopsis thaliana*) [11], 151 in rice (*Oryza sativa*) [11], 163 in poplar (*Populus trichocarpa*) [12], 147 in foxtail millet (*Setaria italica*) [13], 152 in soybean (*Glycine max L*.) [14], 152 in maize (*Zea mays*) [15], 152 in tobacco (*Nicotiana tabacum*) [16], 74 in grapevine (*Vitis vinifera*) [17], 167 in banana (*Musa acuminata*) [18], 104 in tomato (*Solanum lycopersicum*) [19], 96 in cassava (*Manihot esculenta Crantz*) [20], 100 in physic nut (*Jatropha curcas L*.) [21], and 188 in Chinese cabbage (*Brassica rapa*) [22]. NAC TFs are characterized by multi-functionality in regulating vital biological processes, such as shoot apical meristem formation [23, 24], lateral root development [25, 26], secondary wall formation [27], leaf senescence [28–30], and seed development, during the life cycle of plants [31]. In addition, Arabidopsis ANAC078 protein is involved in flavonoid biosynthesis [32].

Numerous NAC genes participate in the response to environmental stresses and hormone signaling. *ANAC019*, *ANAC055* and *ANAC072/RD26* are early-identified and well-characterized stress-related Arabidopsis NAC genes that are induced by drought, salinity, cold and ABA (abscisic acid) [33]. Microarray analysis of transgenic plants overexpressing either of these genes revealed that stress-inducible genes are upregulated and drought tolerance is significantly improved in these plants [33, 34]. Similar to *ANAC072/RD26*, *ATAF1* is another Arabidopsis NAC gene whose overexpression enhances plant tolerance to drought, ABA, salt, oxidative stress, and necrotrophic pathogen [35]. The overexpression of several rice NAC genes significantly enhances tolerance and maintains grain yield [36–38].

Genomic sequencing of tea plant has not been completed. The completed transcriptome sequencing of tea plant provides an opportunity to identify gene families at the transcriptome level [39]. Recent studies have identified and analyzed 89 AP2/ERF, 50 WRKY, 16 HSF, and 18 bZIP genes in tea plant; the roles of these genes in response to adversity stresses were also elucidated [40–43]. To date, detailed analysis of NAC family genes in tea plant remains to be conducted.

In the present study, the transcriptome data was just utilized to extract the NAC sequences of tea plant. A total of 45 CsNAC genes were identified in tea plant based on RNA-Seq data [39]. Subsequently, multiple sequence alignment, conserved motif analysis, phylogenetic construction and membrane-bound proteins identification were performed. On the basis of evolutionary relationships and structural analysis, nine NAC genes were selected for quantitative real-time PCR analysis. The expression levels of these genes were investigated in various tissues (bud, young leaf, mature leaf, young stem and root) and under diverse environmental stresses (drought, salt, heat, cold and ABA) in two cultivars of tea plant, namely, ‘Huangjinya’ and ‘Yingshuang’. This study provides novel insights into the structures and functions of NAC genes in tea plant and serves as a valuable resource for the improvement of plant stress tolerance.

## Materials and Methods

### Identification of NAC gene family in tea plant

The key words “NAC” and “NAM” were used to retrieve potential unigenes from the summary of gene annotations of the tea plant transcriptome [39]. To identify the candidate NAC genes, BLASTp search (http://blast.ncbi.nlm.nih.gov) of the retrieved proteins were performed. Only those proteins with E-values less than 1e^−10^ and NAM domains above 100 were collected for further investigation. The accuracy of several *CsNAC* genes were validated by PCR paired-end sequencing, and completed by the GenScript Corporation (Nanjing, China). The primer designed from the 5’ UTR and 3’ UTR (Table 1).

**Table 1:** Primers used for PCR amplification of *CsNAC* genes.

Target Gene	Forward primer sequence (5'→3')	Reverse primer sequence (5'→3')
*CsNAC16*	GGAGAGACCATTTCAGATTCA	CTGTACACACTACTACACACAACAAC
*CsNAC17*	AAATGGGAGTTTCCGATACGA	CTCCAAAGCCAAAATGTTAGC
*CsNAC26*	ATGGAGAGCACCGATTCGTCG	AGAATACCAATTCAACCCCGT
*CsNAC29*	CTCCAGCTCAAACTCCC	TCCAACCCACCAAAATA
*CsNAC30*	ATGGGAGTTGCAGAAACCGAC	CTGTCGAAGCCTAAATCCTCC
*CsNAC33*	ATGGGTTTTTAAGATGTGTG	GGTTATTCTGATGGGGC
*CsNAC39*	GGGATACACACATTTCGCACA	GTGACAAAACAACTATATGGCTCAT
*CsNAC45*	AACCCTAAAAAACCTCACC	CTAACGATCTCGAACAACC

The Pfam database (http://pfam.sanger.ac.uk) was used, and hidden Markov models (HMMs) of the collected CsNACs were obtained. These models could serve as another proof of the confirmed CsNACs. To obtain the Arabidopsis orthologs of the NAC proteins of tea plant, all valid sequences were subjected to BLASTp search at Arabidopsis protein TAIR10 release (http://www.arabidopsis.org) by using default parameters. Furthermore, TMHHM server v. 2.0 (http://www.cbs.dtu.dk/services/TMHMM/) was used, and the membrane-bound proteins of CsNAC were predicted.

### Multiple sequence alignment, phylogenetic analysis, and conserved motif analysis

Full-length CsNAC protein sequences and three representative ANAC proteins (ANAC019, ANAC055 and ANAC072/RD26) were aligned using the ClustalX 1.83 program with default parameters. To survey the phylogenetic relationships of NAC genes in tea plant, domain sequences of NAC proteins in tea plant and Arabidopsis were used to construct a phylogenetic tree. After multiple alignments were carried out, phylogenetic trees were generated and displayed by the MEGA program (version 5.0) [44] with the following parameters: neighbor-joining (NJ) method, p-distance model, pairwise deletion, and 1000 bootstrap replicates. The Multiple Em for Motif Elicitation (MEME) suite (version 4.10.1) (http://meme-suite.org/tools/meme) was used to identify the conserved motifs, and the default parameters were used, in addition to the maximum number of motifs (10).

### RNA-seq data analysis

To evaluate the expression levels of NAC genes among the different tea plant cultivars, reads per kilobase per million mapped reads (RPKM) were obtained from the RNA-seq database [39]. Transcriptome databases were obtained from several young leaves of four tea plant cultivars, namely, ‘Yunnanshilixiang’ (Tea_T1), ‘Chawansanhao’ (Tea_T2), ‘Ruchengmaoyecha’ (Tea_T3), and ‘Anjibaicha’ (Tea_T4), under normal conditions. The expression cluster of *CsNAC* genes from each cultivar was analyzed and displayed by HemI 1.0 software (http://hemi.biocuckoo.org/faq.php).

### Preparation of plant materials

The tea plant cultivars ‘Yingshuang’ and ‘Huangjinya’ are biennial cutting seedling and were grown in a greenhouse at the Nanjing Agricultural University (Nanjing, China). The artificial climate conditions were 23°C temperature, 14/10 h light/dark, and 70% relative humidity. Tea plants were subjected to various stress treatments. For extreme temperature treatments, the seedlings were maintained at 4°C and 38°C, and some seedlings were irrigated with 20% PEG 6000 and 200 mM NaCl solution, respectively. Additionally, some seedlings were sprayed with 200 μM ABA solution. The young leaves of all tested plants were harvested at 0, 2, 8, and 24 h after treatments, rapidly frozen in liquid nitrogen, and then stored at −80°C.

To study the expression patterns of *CsNAC* genes in different tissues, including bud, young leaf, mature leaf, young stem and root, were collected from growing plants under normal conditions. All harvested samples were rapidly frozen in liquid nitrogen and then stored at −80°C.

### RNA isolation, cDNA synthesis, and qRT-PCR analysis

Total RNA from tea plant materials was extracted using the RNA Isolation Kit (Huyueyang, Beijing, China) in accordance with the manufacture’s instruction. Before cDNA synthesis, RNA integrity was verified using 1.5% denaturing agarose gel, and purity and concentration were measured on a NanoDrop ND-2000 Spectrophotometer (Thermo Scientific, USA). Moreover, the PrimeScript RT reagent Kit (TaKaRa, Dalian, China) was used, and 1 μg of high-quality RNA samples were programed for reverse transcription into cDNA in accordance with the operation manual.

The specific primers used for calculating the relative expression were designed from the nonconserved region of genes using Primer Premier 5.0 software. *Actin* served as a reference gene [45]. The primer sequences used in this experiment are provided in Table 2. The SYBR Premix *Ex Taq* kit (TaKaRa, Dalian, China) was used for qRT-PCR on the Bio-rad IQ5 fluorescence quantitative PCR platform. The experiments were completed in a volume of 20 μL: 0.4 μL of each specific primer, 10 μL of 2 × SYBR Premix, 2 μL of diluted cDNA as a template, and 7.2 μL of ddH_2_O. The reactions were carried out under the following conditions: denaturation at 95°C for 30 s, followed by 40 cycles of 95°C for 10 s and 60°C for 20 s. The experiments were performed with three independent biological replicates, and the relative expression level was calculated using the 2^−ΔΔCT^ method [46].

**Table 2:** Primers for qRT-PCR of *CsNAC* genes.

Target Gene	Forward primer sequence (5'→3')	Reverse primer sequence (5'→3')
*CsNAC1*	ATCGGGTCATACCAGTGCTCT	CGGTTACGAGAAGAGTAAACATAAC
*CsNAC2*	ACACGAAACCAGTAAGGCA	AATGGAGTGTTACCGATAGCA
*CsNAC9*	CTGATTTTGAAGGCTATGGAC	CGATTATGAACTTGGCGAG
*CsNAC12*	CGAGAATGAAGGAGCGAA	AGGTTGGTGGAGGAATGG
*CsNAC15*	TCTTTCGGCTCCTATGCTCAA	CAATGGCGATGAAGAGGCTGT
*CsNAC17*	CCAAAGAACAGAGCCACG	TGGGTATGAAGGAGTTGGG
*CsNAC18*	TAGTTTCTTTCTCCTCACCCTC	TCCTCCCCAAATACATACATAG
*CsNAC26*	ACAAACTACGCCACAATGC	AGGGAGGGTTCTTTTCAGG
*CsNAC29*	ACATCTGGCATTGGTGAAA	ACCAGCAAGGACAACAGC
*CsNAC30*	ATTTCAGGGGTTTCAAGCA	CAGAGAATTCATTCGCGG
*CsNAC32*	ATTCTGAACCACCACGGC	ATTCTGGCGACACGACG
*CsNAC35*	AGAGAGCATGATGGAAAACAGAG	GAAGTAGGCAAAGGGGTGGAGAA
*CsNAC38*	TCTTTCATGGCACTTTCTATGCT	ACTGGACGCACTGTACACTTCTC
*CsNAC44*	GCATCAGTATCACTTTGGCACC	TGACGACTTGCTGTTGCTGAT
*CsNAC45*	GGCTGGGGATGAGATGGTA	CGACACAGAGGTGCCTTGAT
*Actin*	CCATCACCAGAATCCAAGAC	GAACCCGAAGGCGAATAGG

## Results and Discussion

### Identification of NAC family members in tea plant

According to the RNA-seq database, the annotated sequences with “NAC” and “NAM” were searched, and 120 NAC unigenes were obtained. Their corresponding amino acid sequences were subjected to the NCBI BLASTp program. Finally, 45 *CsNAC* genes, designated as *CsNAC1–CsNAC45* (S1 Table), were identified and used for further investigation, although still some of these genes were still fragments. Eight *CsNAC* genes (*CsNAC16*, *CsNAC17*, *CsNAC26*, *CsNAC29*, *CsNAC30*, *CsNAC33*, *CsNAC39* and *CsNAC45*) were examined by PCR-direct sequencing in tea plant cultivar ‘Yingshuang’, and the genes sequences showed very minor discrepancy (S1–S8 Figs). The remaining 75 sequences with an E-value more than 1e^−10^ or a NAM domain length less than 100 were excluded. HMMs were excellent implement in the bioinformatic analysis of biological sequences and the discovery of secondary structures [47]. In the present study, HMMs of the 45 predicted CsNAC proteins were determined using the Pfam database; the results showed that these CsNAC proteins indeed contained a conserved NAM domain (PF02365) at the N-terminus (S2 Table). This result could also be a powerful evidence for the correctness of the valid sequences.

We retrieved the functional annotations of the 45 CsNAC proteins predicted by BLASTX public databases GO, Nt, Nr, COG, Swiss-Prot, and TrEMBL (E-value ≤ 1E-5) (S3 Table). The predicted CsNAC proteins are involved in various abiotic stresses, such as osmotic stress, water deprivation, salt, heat, cold, and wounding, and biotic stresses, such as bacterium, fungus, nematode, and insect. CsNAC proteins are also involved in signal transduction, including various plant hormone signaling pathways, such as ethylene, abscisic acid, jasmonic acid, salicylic acid, and brassinosteroid. In addition, some CsNAC proteins may be implicated in the development of tissues and organs, such as leaf, seed, embryo, ovule, pollen, and lateral root. Moreover, four tea plant NAC proteins, namely, CsNAC2, CsNAC21, CsNAC26, and CsNAC33, are associated with flavonoid biosynthesis. Catechin (flavan-3-ols), a vital secondary metabolite of tea plant, is produced *via* flavonoid biosynthesis [48]. This finding indicates that NAC may be involved in catechin biosynthesis. The above annotations describe the potential functions of NAC proteins in tea plant, and the intensity of functions may be discrepant, which needs further research.

### Phylogenetic analysis of the NAC TFs between tea plant and Arabidopsis

**Fig 1:**
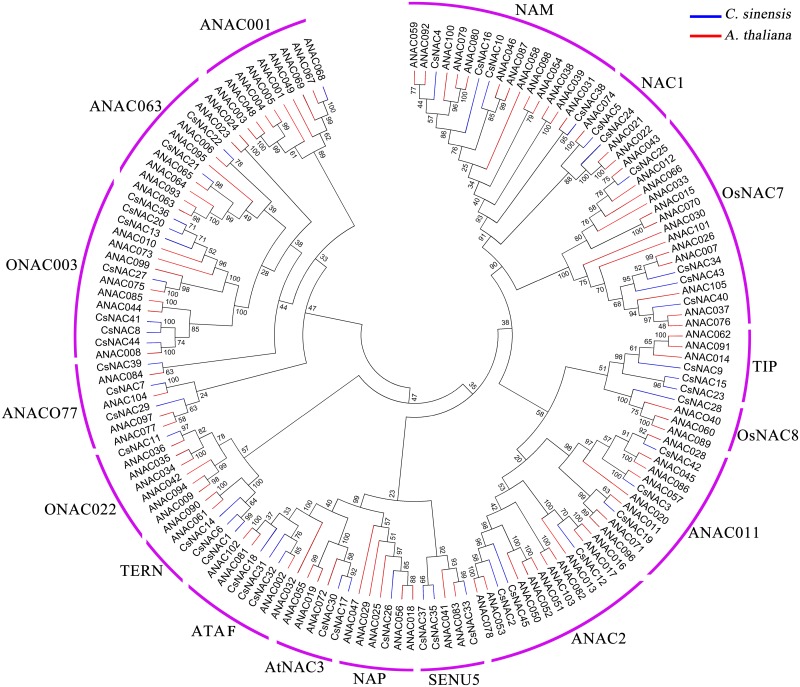
Phylogenetic tree of NAC proteins from *A*. *thaliana* and *C*. *sinensis*. The conserved domain sequences of NAC protein were aligned by Clustal X 1.83, and the phylogenetic tree was constructed using MEGA 5.0 by the neighbor-joining (NJ) method with 1000 bootstrap replicates. The NAC proteins were grouped into 17 distinct subgroups with the pink line. “CsNACs” indicates the NAC proteins from *C*. *sinensis*. “ANACs” represents the NAC proteins from *A*. *thaliana*.

Several NAC genes have been elucidated in Arabidopsis, a typical model plant. To clarify the phylogenetic relationships of NAC family proteins in tea plant and Arabidopsis, an unrooted tree was established on the basis of the aligned NAC domain (A–E) sequences. The results indicated that most of the obtained subgroups were consistent with previous phylogenetic analyses [5, 20]. As shown in Fig 1, the phylogenetic tree clustered all of the NAC members into 17 subgroups. CsNAC proteins were diverse as ANAC proteins, and 45 CsNACs were unevenly distributed in 16 subgroups. By contrast, no member was found in subgroup ANAC001. Notably, the same phenomenon was observed in the phylogenetic analysis of rice and Arabidopsis NAC proteins. All subgroup ANAC001 members belong only to Arabidopsis [5], indicating that this subgroup may either be lost in tea plant and rice or acquired in Arabidopsis after the divergence of their last common ancestor. Furthermore, these NACs may have specialized roles in Arabidopsis. Therefore, the characteristics of this subgroup may be important in researching the genetic relationships among other plant species.

Genes showing a close evolutionary relationship generally exhibit similar functions. Phylogenetic analysis can be utilized to predict gene function, which is important in subsequent functional studies. Predicting stress-related functional genes on the basis of phylogenetic relationships is effective [14, 49]. Subgroups AtNAC3, ATAF, and NAP share a close relationship, and most published stress-related NAC family members are included in these three subgroups. Arabidopsis contains three members (ANAC019, ANAC055 and ANAC072/RD26) of subgroup AtNAC3 and four members (ATAF1/ANAC002, ATAF2/ANAC081, ANAC032 and ANAC102) of subgroup ATAF, all of which are involved in multiple-stress response [9, 33, 50]. However, few reports refer to subgroup NAP for the stress resistance in Arabidopsis until now. The subdomain E of ATAF, AtNAC3 and NAP members is highly conserved and may determine the function of NAC proteins, indicating that subgroup NAP might also be involved in stress resistance [5]. ANAC029 is closely associated with leaf senescence in Arabidopsis [28]. Senescence is a common phenomenon when plants are subject to various serious adversity stresses. A minimum of three NAC genes belong to subgroup NAP in *Chrysanthemum lavandulifolium*, which could be induced by salt, drought, and salicylic acid [51]. These findings indicate that the subdomain NAP belong to the stress-related group. Therefore, we consider CsNAC17 and CsNAC30 from subgroup AtNAC3; CsNAC18, CsNAC31, and CsNAC32 from subgroup ATAF; and CsNAC26 from subgroup NAP as stress-related proteins.

To understand further the characteristics of NAC genes in tea plant, we confirmed that the NAC proteins of Arabidopsis are orthologous to 45 CsNACs (S4 Table). Using a Blastp search, the most satisfactory match of Arabidopsis protein was regarded as orthologous. The score and E-value were used to explain the satisfactory match of orthologous. As showed in S4 Table, CsNAC17, CsNAC29, and CsNAC30 were orthologous to Arabidopsis ANAC072/RD26, of which CsNAC17 and CsNAC30 were highly orthologous to Arabidopsis ANAC072/RD26 with more than 300 scores and powerful E-value support. CsNAC18, CsNAC31 and CsNAC32 were highly orthologous to Arabidopsis ANAC002/ATAF1 with at least 200 scores and powerful E-value support. The homologous relations of tea plant and Arabidopsis considerably matched the phylogenetic analysis. CsNAC29 exhibited a close evolutionary relationship to ANAC096, which functions synergistically with ABF2 and ABF4, and helps plants survive under dehydration and osmotic stresses [52]. Finally, *CsNAC17*, *CsNAC18*, *CsNAC26*, *CsNAC29*, *CsNAC30*, *CsNAC31*, and *CsNAC32* were considered the potential stress-related genes.

### Gene structure and conserved motif analysis

The diversity of plant protein sequence and structure generated in biological evolution is a possible mechanism for the formation of multigene families and functional diversities; the diversity characteristics of plants lead to the efficient use of natural resources or adapt to adverse environments [53]. To understand further the structural features of NAC proteins in tea plant, multiple sequence alignment of full-length CsNAC proteins was performed, and conserved motifs were predicted in concert with their phylogenetic relationships.

**Fig 2:**
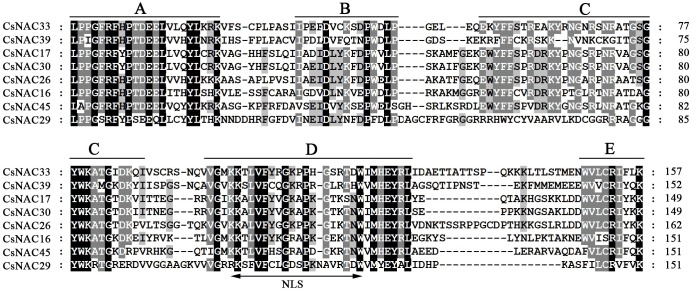
Multiple sequence alignment of eight sequenced CsNAC proteins. Conserved NAC domain is divided into five subdomains (A–E), which are indicated by lines above the sequences. The putative nuclear localization signal (NLS) is shown by a double-headed arrow below the sequences.

To examine the structure of CsNAC proteins, multiple sequence alignment of the full-length CsNAC protein sequences, along with three representative ANAC proteins of Arabidopsis was conducted (S9 Fig). In addition, multi-sequence alignment of eight sequenced CsNAC proteins (CsNAC16, CsNAC17, CsNAC26, CsNAC29, CsNAC30, CsNAC33, CsNAC39 and CsNAC45) was performed (Fig 2). As expected, most of the CsNAC proteins shared a typical highly conserved N-terminal domain, which was divided into five subdomains (A, B, C, D, and E), and a highly variable C-terminal transcriptional regulation domain. The sequence alignments in S9 Fig showed that subdomains A, C, and D were more conserved than subdomains B and E, even though some domain regions were incomplete [5]. In subdomain D, a conserved bipartite nuclear localization signal was identified in most of the CsNAC proteins. This signal has been identified from many NAC proteins in other plant species, indicating these *CsNACs* are nuclearly localized [14, 54, 55].

**Fig 3:**
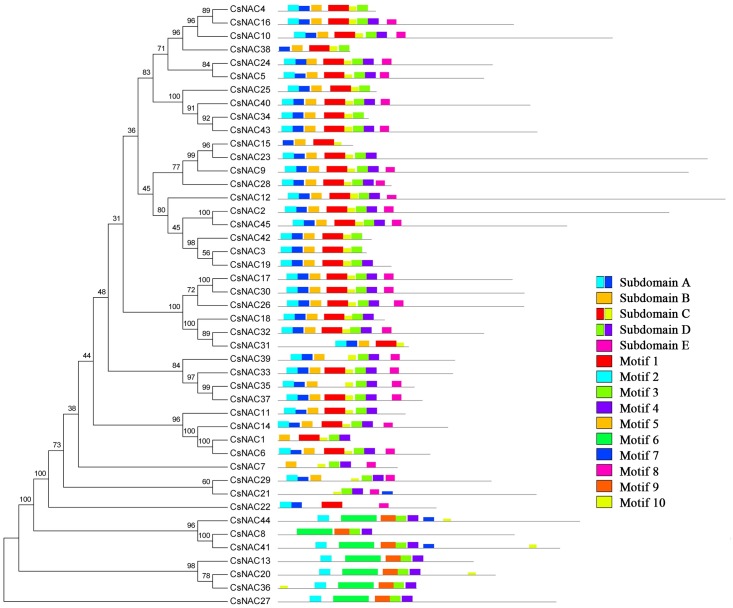
Conserved motif compositions of CsNAC proteins. The unrooted phylogenetic tree was constructed using the conserved domain sequences. The sequences of NAC proteins were aligned by Clustal X 1.83, and the phylogenetic tree was constructed using MEGA 5.0 by the neighbor-joining (NJ) method with 1000 bootstrap replicates. Motifs of NAC proteins were identified by MEME. Each motif is represented by a colored box numbered. Black lines represent non-conserved sequences. The relationships between motifs and conserved domains (A–E) are shown.

To reveal the sequence features of *CsNAC* genes, the conserved motifs were predicted using the MEME program, and 10 conserved motifs were defined (Table 3 and S10 Fig). Members with a close evolutionary relationship displayed uniform or similar motif compositions (Fig 3). In the majority of the CsNAC proteins, motifs 2 plus 7, 5, 1 plus 10, 3 plus 4, and 8 corresponded to the conserved subdomains A, B, C, D and E, respectively. Almost all of the conserved motifs are present at the N-terminal conserved domain region, whereas a motif hardly appears at the diversified C-terminal region of the NAC protein. Previously, 10 putative motifs from potato and physic nut, 15 putative motifs from cassava were predicted; similar, most of the conserved motifs were also observed at the N-terminal NAC domain [20, 21, 55]. Ooka et al. investigated the C-terminal region of the NAC proteins from Arabidopsis and rice; 13 motifs were found, despite the C-terminal region was highly divergent [5]. As a whole, the distribution of the main conserved motifs in tea plant and other species was similar; the different nature of growing habits, such as annual vs perennial and woody vs herbaceous, that may contribute to the differences. The distribution of the conserved motifs indicates that NAC protein functional characteristics may be mainly determined by the N-terminal conserved domain and that C-terminal region may also be involved in determining the functions of these proteins.

**Table 3:** Regular expression levels of conserved motifs from CsNAC proteins.

Motif	E-value	Sites aa	Width	Amino acid sequence composition of motif	NAC Subdomain
1	1.6e-626	33	29	WYFF[SC]P[RK]D[RK]KYP[NT]GSR[TP]NRATG[SA]G[YF]WKAT	C
2	1.4e-367	39	16	SLPPGFRFHP[TS]DEEL[VIL]	A
3	2.1e-328	42	15	[VI]GM[KR]KTLVFYKG[RK]AP	D
4	1.2e-299	36	15	KGE[KR]T[ND]W[VI]MHEYRLx	D
5	6.9e-259	36	15	IP[ED][IV]DLYK[CF][ED]PW[DE]LP	B
6	1.5e-184	7	50	IDEFI[PL]T[VIL][DE][GK]E[DN]GICYTHPE[NKR]LPGV[KS]KDG[SLQ][IS][RV]HFFHR[PT][SI][KN]AY[TA]TGTRKRR	
7	6.8e-093	38	15	x[HY]YLK[RK]K[IV]A[GS]QP[IFL]x[LV]	A
8	1.2e-091	25	13	K[DE][DE][WF]V[LV]CR[IV][FY]KK[SN]	E
9	8.1e-048	7	21	GG[ED][TV]RWHKTGKT[RK]PV[MF][ELV][NG]G[VK][QV]	
10	9.1e-035	41	11	GKD[KR]P[IV]YSxSx	C

### Membrane-associated CsNAC subfamily

**Fig 4:**
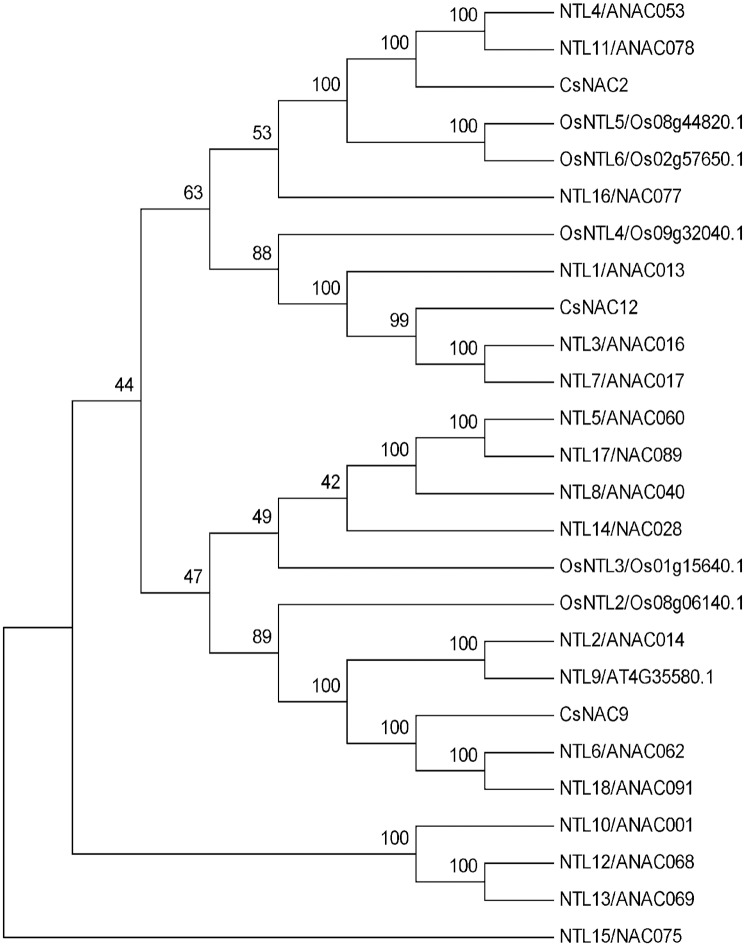
Phylogenetic tree of membrane-bound NACs from *C*. *sinensis*, *A*. *thaliana*, and *O*. *sativa*. The unrooted phylogenetic tree was constructed using full-length NAC MTF sequences. The sequences were aligned by Clustal X 1.83, and the phylogenetic tree was constructed using MEGA 5.0 by the neighbor-joining (NJ) method with 1000 bootstrap replicates.

Recently, membrane-associated TFs (MTFs) have received considerable attention, because of their critical roles in the gene regulatory network of plants [56]. Commonly, these TFs exist in a dormant state; once stimulated, the dormant form becomes activated through proteolytic cleavage; with the degradation of their cytoplasmic anchors, the activated TFs enter the nucleus and regulate the expression of target genes [56, 57]. In the present study, using TMHHM server v. 2.0, we identified three CsNAC proteins (CsNAC2, CsNAC9, and CsNAC12) to contain a single α-helical TM at C-terminal ends (S5 Table). TM commonly exists in highly variable regions of the C-terminus, indicating that many potential MTFs are existing. Sufficient evidence has indicated that NAC MTFs play important roles in plant growth and development as well as in response to abrupt environmental changes [57, 58]. All of the MTF NAC genes in Arabidopsis and rice could be induced by at least one type of environmental stress, namely, drought, salt, cold or heat [9, 59]. The phylogenetic relationship of NAC MTFs among tea plant, Arabidopsis and rice showed that CsNAC MTFs have a close relationship with Arabidopsis MTFs. Thus, NAC MTFs in tea plant might have similar functions to those in Arabidopsis (Fig 4). The finding indicates that CsNAC MTFs may be involved in stress response.

**Fig 5:**
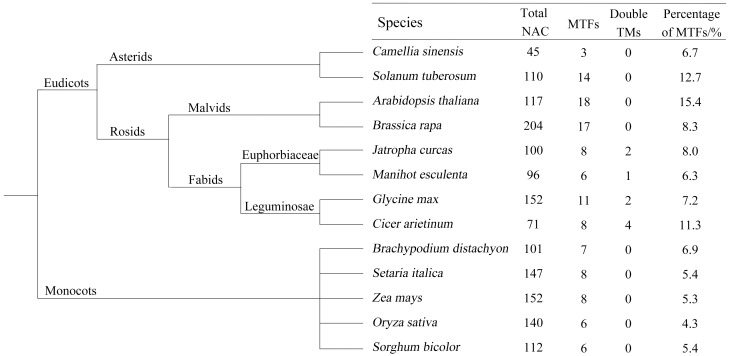
Summary of NAC MTFs in plants.

Several NAC MTFs were identified among various species through intensive genome-wide analysis. We investigated the NAC TFs whose MTFs have been analyzed in whole genomes and 12 species were collected (Fig 5), including *A*. *thaliana* [58], *O*. *sativa* [58], *G*. *max* [14], *Solanum tuberosum* [55], *Sorghum bcolor* [60], *S*. *italic* [13], *B*. *rapa* [61], *Cicer arietinum* [62], *Zea mays* [15], *Brachypodium distachyon* [63], *M*. *esculenta* [20], and *J*. *curcas* [21]. The NAC genes of some species were not used for analysis. For example, 104 NAC genes in *S*. *lycopersicum* [19], 145 in *G*. *raimondii* [53] and 74 in *V*. *vinifera* [17] were identified in genome-wide analysis, but the NAC MTFs were not analyzed. All of the identified NAC MTFs belonged to monocots and eudicots, although some NAC genes had been found in moss and lycophyte in previous studies. Obviously, the total number of NAC genes significantly differs in different species and may mainly undergo paleopolyploidy. For example, Chinese cabbage has undergone triplication since its divergence from Arabidopsis [64]. The proportion of NAC MTFs in monocots was obviously less than that in eudicots. Ha et al. [62] showed that only *G*. *max* and *C*. *arietinum* possess two TMs, suggesting that the existence of doubled TMs is specific to leguminous plants. In the present study, Euphorbiaceae (*M*. *esculenta* and *J*. *curcas*) also possessed doubled TMs. Coincidentally, Euphorbiaceae and leguminous plants could be classified in fabids, suggesting that the existence of doubled TMs is wide and specific to fabids plants. The finding indicates that NAC MTFs are closely related to plant evolution.

### Expression profiles of *CsNAC* genes in four tea plant cultivar transcriptomes

**Fig 6:**
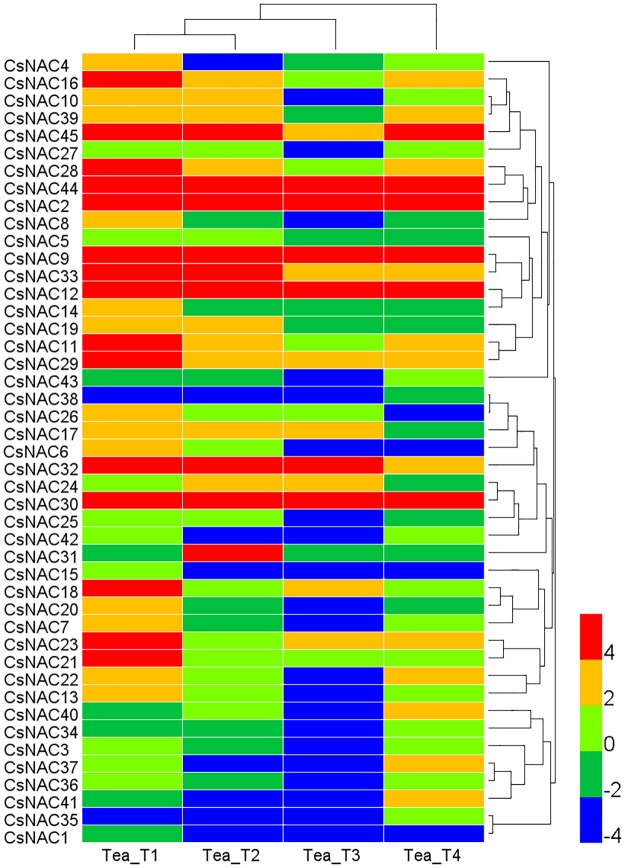
Heat map representation and hierarchical clustering of *CsNAC* genes among four tea cultivars under non-stress conditions. The FPKM values of the Illumina RNA-seq data were reanalyzed and log2 transformed, and heat map were generated using HemI 1.0 software. Bar at the bottom right corner represents log2 transformed values, and bar notes represent different expression levels (red represents highest expression, and blue represents lowest expression).

Illumina RNA-seq data were obtained to assess the expression profiles among different tea plant cultivars of *CsNAC* genes under non-stress conditions [39]. The transcript abundance of 45 *CsNAC* genes was assessed in accordance with the RPKM values of four tea plant cultivars, namely, ‘Yunnanshilixiang’ (Tea_T1), ‘Chawansanhao’ (Tea_T2), ‘Ruchengmaoyecha’ (Tea_T3), and ‘Anjibaicha’ (Tea_T4), although some genes were lowly or barely expressed in some tea plant cultivars (S6 Table). A heat map was displayed on the basis of log2 transformed RPKM values (Fig 6). *CsNAC2*, *CsNAC9*, *CsNAC12*, *CsNAC30*, and *CsNAC44* genes displayed high and stable expression levels in all of the tea plant cultivars. By contrast, *CsNAC1* and *CsNAC38* were barely expressed. Remarkably, *CsNAC2*, *CsNAC9*, and *CsNAC12* belonged to NAC MTFs. The gene expression levels are highly associated with gene function, which indicates that NAC MTFs play important roles in the growth and development of tea plant. In addition, some genes showed obvious cultivar specificity. For example, *CsNAC31* was highly expressed in Tea_T2, but weakly expressed in the three other tea plant cultivars. *CsNAC4*, *CsNAC7*, and *CsNAC40* completely differed among the four tea plant cultivars. Twenty *CsNAC* genes obviously differed among three tea plant cultivars. In Tea_T1, *CsNAC* genes showed high abundance. Sixteen genes were highly expressed among at least one tea plant cultivar, and only one gene was not expressed in Tea_T1. In Tea_T3, *CsNAC* genes showed relatively low expression levels. Seventeen *CsNAC* genes were not detected in at least one tea plant cultivar, and all of them happened in Tea_T3; while Tea_T2 had four, and Tea_T1 and Tea_T4 only had one (S6 Table). In summary, the RPKM values revealed that the four tea plant cultivars differed in the expression of *CsNAC* genes. These tea plant cultivars are grown in different regions of China [39, 40], and the differences may occur on the individual evolution of different cultivars.

### Expression profiles of CsNAC genes in different tissues of tea plant

**Fig 7:**
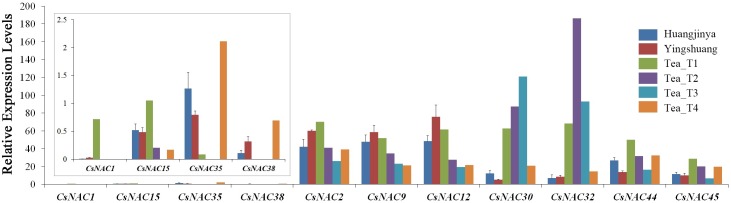
Comparison of the expression profiles of four low expression *CsNAC* genes and seven high expression genes among six tea plant cultivars determined by RNA-Seq and qRT-PCR. The separate box was magnified expression profiles of four low expression *CsNAC* genes. Tea_T1, Tea_T2, Tea_T3, and Tea_T4 were determined by RNA-Seq; ‘Huangjinya’ and ‘Yingshuang’ were determined by qRT-PCR. Bars represent the mean values of three replicates ± standard deviation (SD).

‘Huangjinya’ and ‘Yingshuang’ are two of the widely planted tea plant cultivars in China. ‘Huangjinya’, a light-sensitive albino tea plant cultivar, has a potential for processing high quality green tea even in summer, but it shows weak stress resistance [65]. ‘Yingshuang’, a tea plant cultivar with good resistance to abiotic stresses, especially cold stress, possesses considerable agronomic traits, such as appropriate phenol ammonia content and good cold resistance. Based on the above analysis of the transcriptome, four low expression genes (*CsNAC1*, *CsNAC15*, *CsNAC35* and *CsNAC38*) and seven high expression genes (*CsNAC2*, *CsNAC9*, *CsNAC12*, *CsNAC30*, *CsNAC32*, *CsNAC44* and *CsNAC45*) were selected for verification through qRT-PCR analysis in two tea plant cultivars, ‘Huangjingya’ and ‘Yingshuang’ (Fig 7). The four low expression genes were still barely expressed, and the seven high expression genes displayed high and stable expression levels in two tea plant cultivars, ‘Huangjingya’ and ‘Yingshuang’.

NAC genes are involved in various developmental processes [17, 66]. As natural properties of each gene, tissue-specific expression is closely associated with the growth and development of the particular tissue. In the present study, nine *CsNAC* genes, including six predicted stress-related and three MTF *CsNAC* genes, were selected for subsequent qRT-PCR analysis. The expression patterns of these genes were assessed in five tissues, including root, young stem, mature leaf, young leaf and bud in two tea plant cultivars, namely, ‘Huangjinya’ and ‘Yingshuang’.

**Fig 8:**
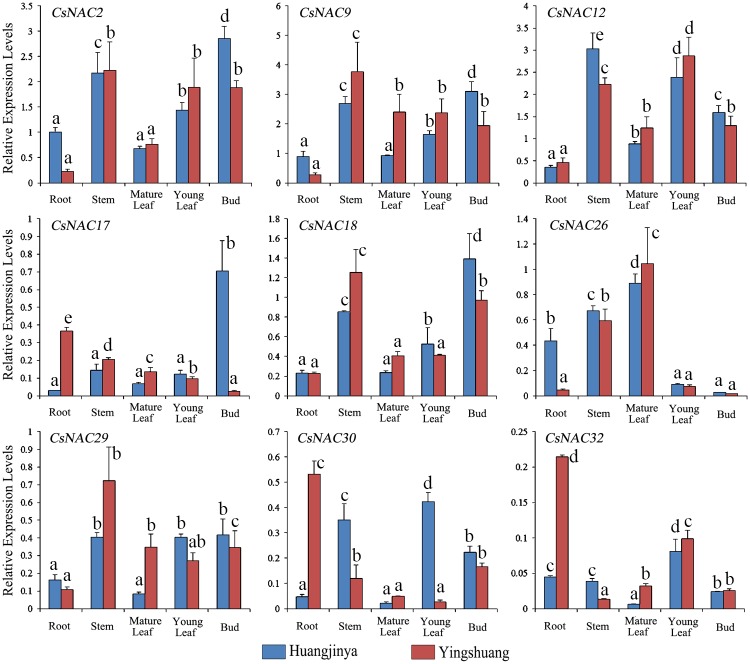
Expression profiles of selected *CsNAC* genes in different tissues of two tea plant cultivars, ‘Huangjinya’ and ‘Yingshuang’. Bars represent the mean values of three replicates ± standard deviation (SD). Different small letters indicate significant differences at *P* < 0.05.

These nine NAC genes widely exist in the five tissues of the two tea cultivars, and the expression patterns varied (Fig 8). Compared with other genes, three MTF *CsNAC* genes, namely, *CsNAC2*, *CsNAC9*, and *CsNAC12*, exhibited higher expression levels in all of the tissues. This result is consistent with the above expression analysis among different cultivars, suggesting the critical role of the genes in plant growth and development. In general, the same NAC genes exhibited similar tissue-specific expression profiles in the two tea cultivars. For example, in the two tea plant cultivars, *CsNAC2* and *CsNAC18* were highly expressed in young stem and bud, *CsNAC12* in young stem and young leaf, and *CsNAC26* in stem and mature leaf. However, *CsNAC26* was lowly expressed in young leaf and bud. Discrepant expression patterns were also observed. For example, *CsNAC2*, *CsNAC9*, and *CsNAC26* showed higher expression level in the root of ‘Huangjinya’ than in that of ‘Yingshuang’; *CsNAC17* showed the highest expression level in the root of ‘Yingshuang’ and in the bud of ‘Huangjinya’; *CsNAC30* and *CsNAC32* showed higher expression levels in the root of ‘Yingshuang’ than in that of ‘Huangjinya’.

Tissue-specific genes facilitate the development of particular tissues. *NAC1* and *ANAC2*, which are overexpressed in the roots of transgenic Arabidopsis, promote the development of lateral roots [25, 67]. Arabidopsis *ANAC036*, which is strongly expressed in the leaves of transgenic plants, shows a semi-dwarf phenotype [68]. In general, the tissue expression profiles of NAC genes in different cultivars provide an evidence for further investigation on the development of tea plant.

### Expression profiles of *CsNAC* genes under various stress treatments in tea plant

Environment stress adversely affect plant growth and productivity, and trigger a series of morphological, physiological, biochemical and molecular changes [69]. Considerable evidence has shown that NAC genes play vital roles in the response to biotic or abiotic stresses [9, 70]. To explore the roles of NAC genes in tea plant under diverse environmental conditions, nine *CsNAC* genes, including six stress-related and three MTF *CsNAC* genes, were selected and subjected to qRT-PCR expression analysis in response to multiple stress treatments in ‘Huangjinya’ and ‘Yingshuang’. Such treatments include drought (20% PEG 6000), salinity (200 mM NaCl), heat (38°C), cold (4°C) and ABA (200 μM).

#### Drought treatment

**Fig 9:**
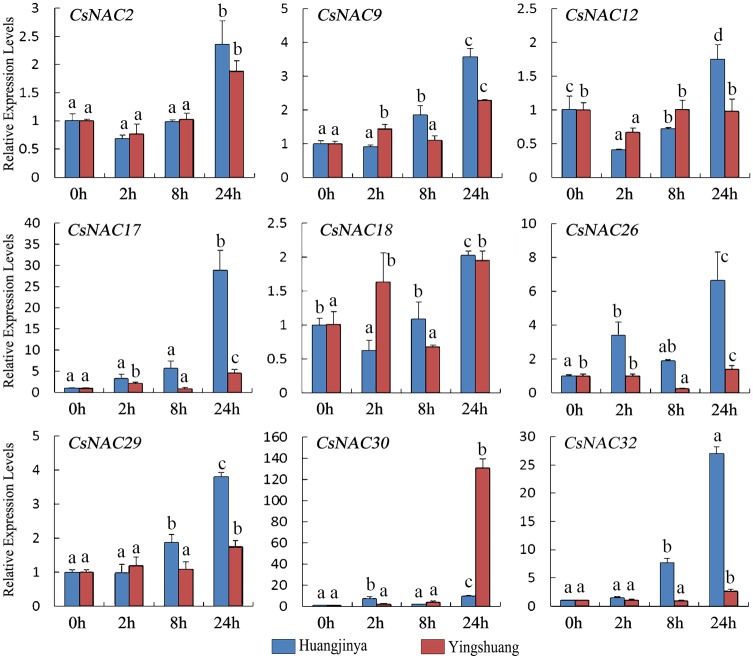
Expression profiles of *CsNAC* genes in tea plant cultivars ‘Huangjinya’ and ‘Yingshuang’ under drought stress. Bars represent the mean values of three replicates ± standard deviation (SD). Different small letters indicate significant differences at *P* < 0.05.

Under drought treatment (Fig 9), most of the *CsNAC* genes were upregulated in both tea plant cultivars. The relative transcript levels of seven *CsNAC* genes (*CsNAC2*, *CsNAC9*, *CsNAC12*, *CsNAC17*, *CsNAC29*, *CsNAC30*, and *CsNAC32*) gradually increased and reached the highest level at 24 h. *CsNAC2* and *CsNAC12* were initially downregulated. *CsNAC18* reached a maximum of about two-fold despite the decrease at 2 and 8 h in ‘Huangjinya’ and ‘Yingshuang’, respectively. *CsNAC26* was upregulated at all time points in ‘Huangjinya’ but was relatively stably expressed in ‘Yingshuang’ despite the decrease at 8 h. Notably, *CsNAC17*, *CsNAC30* (ortholog of *ANAC072/RD26*), and *CsNAC32* (ortholog of *ANAC002/ATAF1*) were highly induced (over 20-fold) in at least one tea plant cultivar, indicating their possible roles in drought stress responses.

#### Salt treatment

**Fig 10:**
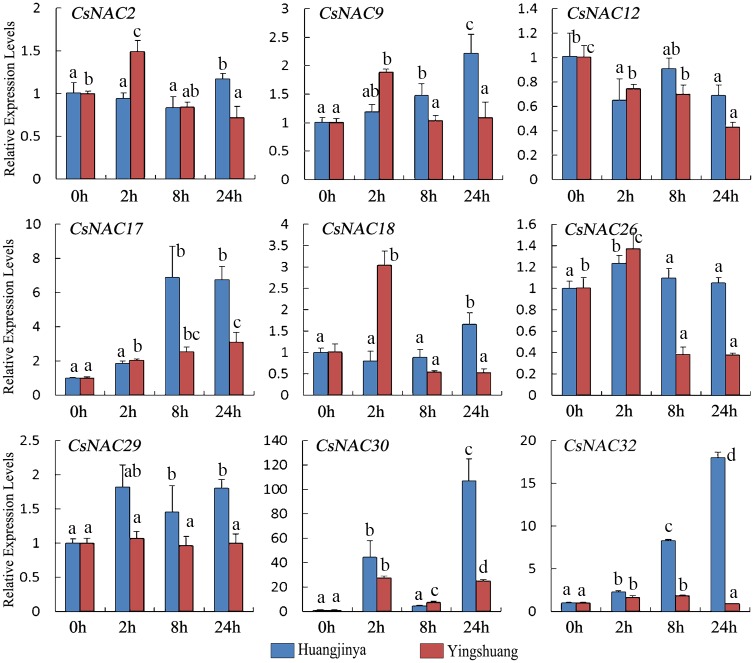
Expression profiles of *CsNAC* genes in tea plant cultivars ‘Huangjinya’ and ‘Yingshuang’ under salt stress. Bars represent the mean values of three replicates ± standard deviation (SD). Different small letters indicate significant differences at *P* < 0.05.

Under salt treatment (Fig 10), four *CsNAC* genes (*CsNAC9*, *CsNAC17*, *CsNAC30*, and *CsNAC32*) were mainly upregulated in both tea plant cultivars. By contrast, *CsNAC12* exhibited a slight decline. *CsNAC2* showed a relatively stable expression level. *CsNAC26* in ‘Huangjinya’ and *CsNAC29* in ‘Yingshuang’ were also relatively stably expressed. Meanwhile, *CsNAC26* decreased after 8 h in ‘Yingshuang’, and *CsNAC29* slightly increased in ‘Huangjinya’. *CsNAC18* was significantly upregulated in ‘Yingshuang’ at 2 h. *CsNAC30* (ortholog of *RD26*) was the most induced by salt treatment with 107- and 28-fold induction in ‘Huangjinya’ and ‘Yingshuang’, respectively. However, this gene was declined at 8 h and reached another peak at 24 h in both tea plant cultivars, indicating the complexity of gene regulatory networks.

#### High temperature treatment

**Fig 11:**
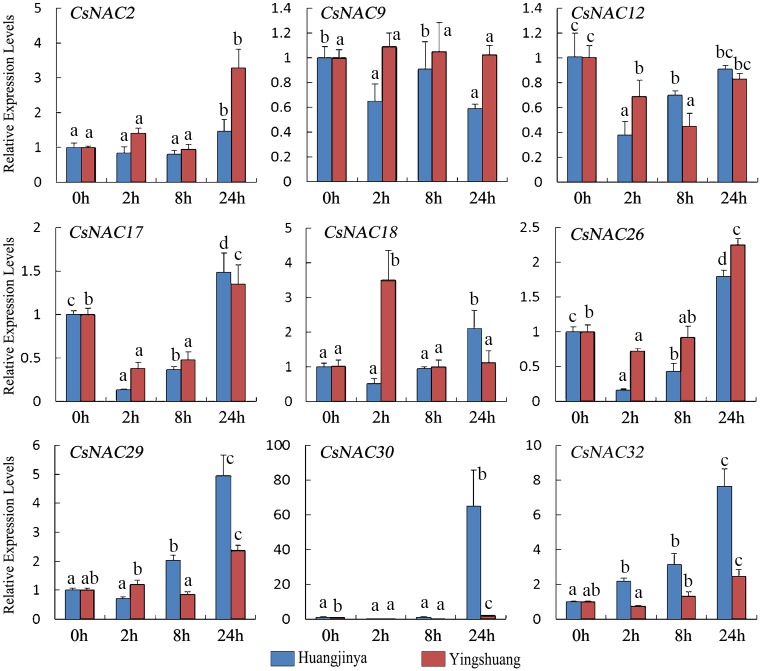
Expression profiles of *CsNAC* genes in tea plant cultivars ‘Huangjinya’ and ‘Yingshuang’ under heat stress. Bars represent the mean values of three replicates ± standard deviation (SD). Different small letters indicate significant differences at *P* < 0.05.

Under high temperature treatment (Fig 11), seven *CsNAC* genes (*CsNAC2*, *CsNAC17*, *CsNAC26*, *CsNAC29*, *CsNAC30*, and *CsNAC32*) gradually increased and reached the highest level at 24 h. By contrast, *CsNAC17*, *CsNAC26*, and *CsNAC30* were initially downregulated. *CsNAC12* decreased in both tea plant cultivars. *CsNAC9* also slightly decreased in ‘Huangjinya’ but showed a more stable expression in ‘Yingshuang’. After 2 h, *CsNAC18* was gradually upregulated in ‘Huangjinya’ but downregulated in ‘Yingshuang’.

#### Low temperature treatment

**Fig 12:**
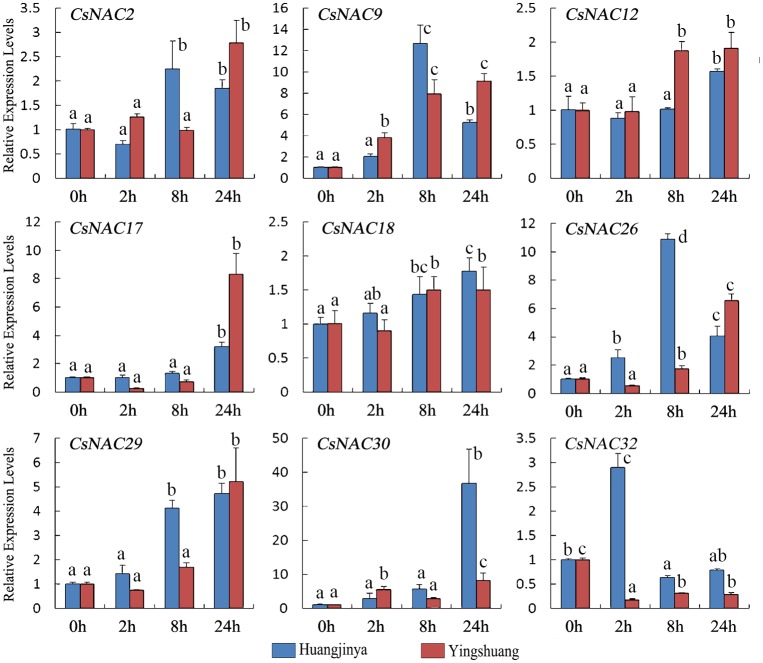
Expression profiles of *CsNAC* genes in tea cultivars ‘Huangjinya’ and ‘Yingshuang’ under cold stress. Bars represent the mean values of three replicates ± standard deviation (SD). Different small letters indicate significant differences at *P* < 0.05.

Low temperature is a type of abiotic intimidation that significantly affects tea plant growth and productivity, particularly the cold of the late spring or late frost [2, 69]. Under cold treatment (Fig 12), *CsNAC32* was downregulated in both tea plant cultivars despite the high expression at 2 h in ‘Huangjinya’. All of the other *CsNAC* genes gradually increased and reached the highest level at 12 or 24 h. Nevertheless, *CsNAC17* and *CsNAC26* were initially downregulated in ‘Yingshuang’. *CsNAC12* and *CsNAC18* slightly increased with less than two-fold in both tea plant cultivars.

In general, most *CsNAC* genes were affected by drought, salinity, and high and cold stresses. Although the intensity of stress responses was varied, most *CsNAC* genes exhibited a similar tendency in the two tea plant cultivars. Whole-genome expression analysis in Arabidopsis showed that most of the NAC genes are responsive to salt and extreme temperatures [8, 71]. *SiNAC* expression profiles showed that all the collected genes display varied expression patterns in response to one or more stresses and that cold stress induces relatively more dramatic changes in transcript abundance than dehydration or salinity [13]. The varied gene expression profiles suggest that *CsNACs* control a complex gene regulatory network and exert a regulatory effect on various physiologic functions for acclimatizing multiple challenges. Moreover, the expression levels of Arabidopsis *RD26* orthologs *StNAC072* and *StNAC101* were highly induced by stress and ABA treatments [55]. Arabidopsis *RD26* orthologs *CaNAC06* and *CaNAC67* are highly induced by dehydration [62], and *MeNAC22* (*RD26* orthologs) is strongly induced by osmotic and drought stresses [20]. Arabidopsis *RD26* orthologs *CsNAC17* and *CsNAC30* were highly induced by at least one stress, which agrees with previous reports. The finding indicates that other species of Arabidopsis *RD26* orthologs are most likely highly induced by stresses. Some research indicated that TF genes that are highly induced by stress could be preferentially utilized for further plant functional studies because of their potential in the development of improved stress-tolerant transgenic plants *via* overexpression. *CsNAC17* and *CsNAC30* were exhibited to be appropriate candidate genes for further plant research in tea plant.

#### ABA treatment

ABA is a plant hormone that not only participates in plant growth and development, but also plays a crucial role in the regulation of various stress responses [72]. NAC genes may be regulated by ABA-dependent or ABA-independent pathways because of the difference in their promoter elements [8, 9, 71, 73].

**Fig 13:**
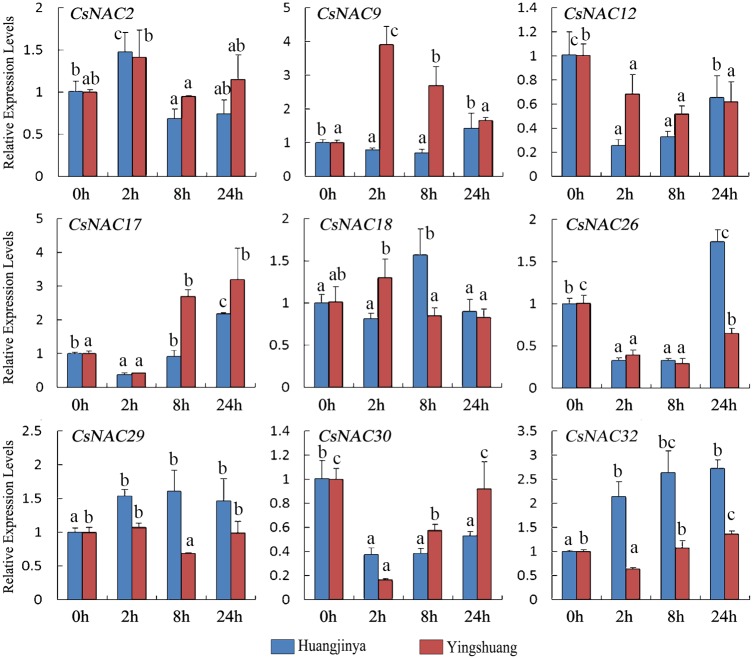
Expression profiles of *CsNAC* genes in tea plant cultivars ‘Huangjinya’ and ‘Yingshuang’ under ABA treatment. Bars represent the mean values of three replicates ± standard deviation (SD). Different small letters indicate significant differences at *P* < 0.05.

**Fig 14:**
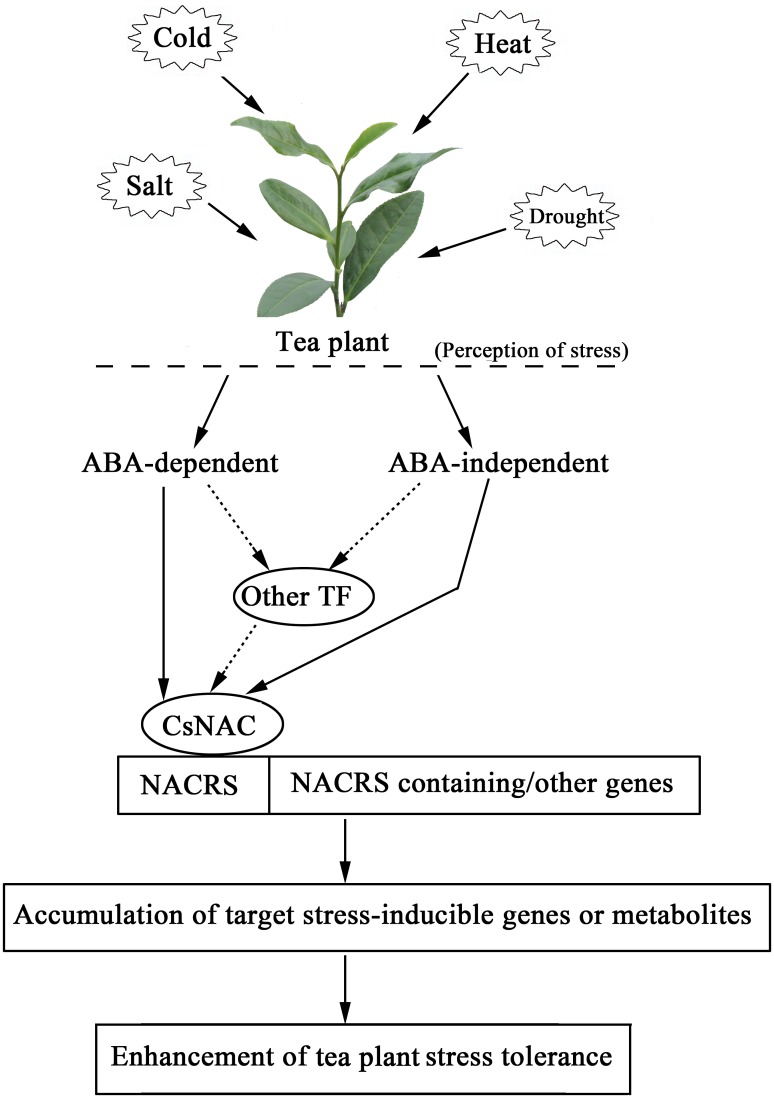
A potential model of transcriptional regulation of NAC TFs under abiotic stress condition in tea plant.

Under ABA treatment (Fig 13), *CsNAC17* initially decreased and then increased in both tea plant cultivars. *CsNAC12*, *CsNAC26*, and *CsNAC30* were mainly downregulated, but *CsNAC26* was upregulated in ‘Huangjinya’ at 24 h. *CsNAC9* was upregulated in both tea plant cultivars, which reached a peak in ‘Yingshuang’ at 24 h and in ‘Huangjinya’ at 2 h. *CsNAC32* was upregulated in ‘Huangjinya’ but was relatively stably expressed in ‘Yingshuang’. The above six *CsNAC* genes responded to at least one stress, indicating that these genes may regulate stress responses in tea plant in an ABA-dependent manner. In addition, *CsNAC2*, *CsNAC18*, and *CsNAC29* showed relatively stable expression levels in both tea plant cultivars. Therefore, *CsNAC2*, *CsNAC18*, and *CsNAC29* may be regulated in ABA-independent pathways. Hu and his colleagues identified that 19 *CaNAC* genes are responsive to dehydration; of these genes, 7 are ABA independent and 12 are ABA dependent [62]. The response of *CsNAC* genes to ABA treatment suggests that these genes play roles in ABA signaling (Fig 14).

## Conclusions

Genomic sequencing of tea plant has not been completed. Thus, transcriptome sequencing has a great potential to discover and identify novel genes and will accelerate the understanding of the regulatory functions of NAC TFs. On the basis of transcriptome sequences, 45 *CsNAC* genes were identified. Phylogenetic analysis, as well as the identification and analysis of motif and MTF members, provided an insight into the functional diversity of CsNAC proteins. The comparative analysis of CsNACs with their corresponding Arabidopsis orthologs helped predict the potential functions of CsNAC proteins. The cultivar and tissue expression profiles of *CsNAC* genes under normal growth conditions were surveyed. Furthermore, the expression analysis of certain *CsNAC* genes during various treatments, such as drought, salinity, heat, cold, and ABA, established substantial evidence to select candidate stress-resistant genes, which could be preferentially utilized to develop tea plants with improved resistance under stress conditions. Tea plant suffers from numerous abiotic stresses, such as heat, cold, drought and salinity (Fig 14). Through either ABA-dependent or -independent manner, CsNAC TFs could regulate downstream target genes, and accumulate stress-inducible genes or metabolites, which enhance tea plant stress tolerance and the adaptation to different kinds of adverse conditions.

## Supporting Information

S1 Fig Comparison of *CsNAC16* gene sequences determined by RNA-Seq and PCR sequencing. The gene sequence from Tea_T1 was determined by RNA-Seq; the gene sequence from ‘Yingshuang’ was determined by PCR sequencing.
(DOC)

S2 Fig Comparison of *CsNAC17* gene sequences determined by RNA-Seq and PCR sequencing. The gene sequence from Tea_T1 was determined by RNA-Seq; the gene sequence from ‘Yingshuang’ was determined by PCR sequencing.
(DOC)

S3 Fig Comparison of *CsNAC26* gene sequences determined by RNA-Seq and PCR sequencing. The gene sequence from Tea_T2 was determined by RNA-Seq; the gene sequence from ‘Yingshuang’ was determined by PCR sequencing.
(DOC)

S4 Fig Comparison of *CsNAC29* gene sequences determined by RNA-Seq and PCR sequencing. The gene sequence from Tea_T2 was determined by RNA-Seq; the gene sequence from ‘Yingshuang’ was determined by PCR sequencing.
(DOC)

S5 Fig Comparison of *CsNAC30* gene sequences determined by RNA-Seq and PCR sequencing. The gene sequence from Tea_T2 was determined by RNA-Seq; the gene sequence from ‘Yingshuang’ was determined by PCR sequencing.
(DOC)

S6 Fig Comparison of *CsNAC33* gene sequences determined by RNA-Seq and PCR sequencing. The gene sequence from Tea_T3 was determined by RNA-Seq; the gene sequence from ‘Yingshuang’ was determined by PCR sequencing.
(DOC)

S7 Fig Comparison of *CsNAC39* gene sequences determined by RNA-Seq and PCR sequencing. The gene sequence from Tea_T4 was determined by RNA-Seq; the gene sequence from ‘Yingshuang’ was determined by PCR sequencing.
(DOC)

S8 Fig Comparison of *CsNAC45* gene sequences determined by RNA-Seq and PCR sequencing. The gene sequence from Tea_T4 was determined by RNA-Seq; the gene sequence from ‘Yingshuang’ was determined by PCR sequencing.
(DOC)

S9 Fig Multiple sequence alignment of 45 identified CsNAC proteins and three representative Arabidopsis NAC proteins. Conserved NAC domain is divided into five subdomains (A–E), which are indicated by lines above the sequences. The putative nuclear localization signal (NLS) is shown by a double-headed arrow below the sequences.
(PNG)

S10 Fig Sequence logos of conserved motifs identified in CsNAC proteins. (PNG)

S1 Table List of the NAC protein sequences in the tea plant transcriptome. (XLS)

S2 Table A catalog of NAC proteins in tea plant with their HMM profiles. (XLS)

S3 Table Functional annotations of NAC transcription factors in tea plant. (XLS)

S4 Table List of NAC transcription factor in tea plant along with their corresponding Arabidopsis orthologs. (XLS)

S5 Table Putative membrane-bound tea plant NACs. (XLS)

S6 Table List of RPKM values of 45 CsNAC TFs in four tea plant cultivars. (XLS)

## Abbreviations

ABA: Abscisic acid
BLAST: Basic Local Alignment Search Tool
HMM: Hidden Markov Model
MEGA: Molecular Evolutionary Genetics Analysis
MEME: Multiple Em for Motif Elicitation
MTF: Membrane-associated transcription factor
NAC: NAM-ATAF1/2-CUC
NACRS: NAC recognition sequence
NCBI: National Center for Biotechnology Information
NJ: Neighbor-joining
qRT-PCR: Quantitative real time polymerase chain reaction
RPKM: Reads per kilobase per million mapped reads
RNA-Seq: RNA sequencing
TF: Transcription factor
TM: Transmembrane motif

## References

[1] Chen Y, Yu M, Xu J, Chen X, Shi J. Differentiation of eight tea (*Camellia sinensis*) cultivars in China by elemental fingerprint of their leaves. Journal of the Science of Food and Agriculture. 2009;89(14):2350–5. 10.1002/jsfa.3716

[2] Vyas D, Kumar S. Tea (*Camellia sinensis* (L.) O. Kuntze) clone with lower period of winter dormancy exhibits lesser cellular damage in response to low temperature. Plant Physiology and Biochemistry. 2005;43(4):383–388. 10.1016/j.plaphy.2005.02.016 15907690

[3] Das A, Das S, Mondal TK. Identification of differentially expressed gene profiles in young roots of tea [*Camellia sinensis* (L.) O. Kuntze] subjected to drought stress using suppression subtractive hybridization. Plant Molecular Biology Reporter. 2012;30(5):1088–1101. 10.1007/s11105-012-0422-x

[4] Yamaguchishinozaki K, Shinozaki K.Transcriptional regulatory networks in cellular responses and tolerance to dehydration and cold stresses. Annual Review of Plant Biology. 2006;57(10):781–803. 10.1146/annurev.arplant.57.032905.105444 16669782

[5] Ooka H, Satoh K, Doi K, Nagata T, Otomo Y, Murakami K, et al Comprehensive analysis of NAC family genes in *Oryza sativa* and *Arabidopsis thaliana*. DNA Res. 2003; 10:239–247. 15029955 10.1093/dnares/10.6.239

[6] Ernst HA, Olsen AN, Larsen S, Lo Leggio L. Structure of the conserved domain of ANAC, a member of the NAC family of transcription factors. EMBO Reports. 2004;5(3):297–303. 10.1038/sj.embor.7400093 15083810 PMC1299004

[7] Chen Q, Wang Q, Xiong L, Lou Z. A structural view of the conserved domain of rice stress-responsive NAC1. Protein & Cell. 2011;2(1):55–63. 10.1007/s13238-011-1010-9 21337010 PMC4875291

[8] Jensen MK, Kjaersgaard T, Nielsen MM, Galberg P, Petersen K, O'Shea C, et al The *Arabidopsis thaliana* NAC transcription factor family: structure-function relationships and determinants of ANAC019 stress signalling. The Biochemical Journal. 2010;426(2):183–196. 10.1042/BJ20091234 19995345

[9] Puranik S, Sahu PP, Srivastava PS, Prasad M. NAC proteins: regulation and role in stress tolerance. Trends in Plant Science. 2012;17(6):369–81. 10.1016/j.tplants.2012.02.004 22445067

[10] Puranik S, Bahadur RP, Srivastava PS, Prasad M. Molecular cloning and characterization of a membrane associated NAC family gene, SiNAC from foxtail millet [*Setaria italica* (L.) P. Beauv]. Molecular Biotechnology. 2011;49(2):138–50. 10.1007/s12033-011-9385-7 21312005

[11] Nuruzzaman M, Manimekalai R, Sharoni AM, Satoh K, Kondoh H, Ooka H, et al Genome-wide analysis of NAC transcription factor family in rice. Gene. 2010;465(1–2):30–44. 10.1016/j.gene.2010.06.008 20600702

[12] Hu R, Qi G, Kong Y, Kong D, Gao Q, Zhou G. Comprehensive analysis of NAC domain transcription factor gene family in *Populus trichocarpa*. BMC Plant Biology. 2010;10:145 10.1186/1471-2229-10-145 20630103 PMC3017804

[13] Puranik S, Sahu PP, Mandal SN, B VS, Parida SK, Prasad M. Comprehensive genome-wide survey, genomic constitution and expression profiling of the NAC transcription factor family in foxtail millet (*Setaria italica* L.). PloS ONE. 2013;8(5):e64594 10.1371/journal.pone.0064594 23691254 PMC3654982

[14] Balazadeh S, Kwasniewski M, Caldana C, Mehrnia M, Zanor MI, Xue GP, et al ORS1, an H(2)O(2)-responsive NAC transcription factor, controls senescence in *Arabidopsis thaliana*. Molecular Plant. 2011;4(2):346–60. 10.1093/mp/ssq080 21303842 PMC3063519

[15] Shiriga K, Sharma R, Kumar K, Yadav SK, Hossain F, Thirunavukkarasu N. Genome-wide identification and expression pattern of drought-responsive members of the NAC family in maize. Meta Gene. 2014;2:407–17. 10.1016/j.mgene.2014.05.001 25606426 PMC4287890

[16] Rushton PJ, Bokowiec MT, Han S, Zhang H, Brannock JF, Chen X, et al Tobacco transcription factors: novel insights into transcriptional regulation in the Solanaceae. Plant Physiology. 2008;147(1):280–95. 10.1104/pp.107.114041 18337489 PMC2330323

[17] Wang N, Zheng Y, Xin H, Fang L, Li S. Comprehensive analysis of NAC domain transcription factor gene family in Vitis vinifera. Plant Cell Reports. 2013;32(1):61–75. 10.1007/s00299-012-1340-y 22983198

[18] Cenci A, Guignon V, Roux N, Rouard M. Genomic analysis of NAC transcription factors in banana (*Musa acuminata*) and definition of NAC orthologous groups for monocots and dicots. Plant Molecular Biology. 2014;85(1–2):63–80. 10.1007/s11103-013-0169-2 24570169 PMC4151281

[19] Su H, Zhang S, Yin Y, Zhu D, Han L. Genome-wide analysis of NAM-ATAF1,2-CUC2 transcription factor family in *Solanum lycopersicum*. Journal of Plant Biochemistry and Biotechnology. 2014;24(2):176–83. 10.1007/s13562-014-0255-9

[20] Hu W, Wei Y, Xia Z, Yan Y, Hou X, Zou M, et al Genome-wide identification and expression analysis of the nac transcription factor family in Cassava. PloS ONE. 2015;10(8):e0136993 10.1371/journal.pone.0136993 26317631 PMC4552662

[21] Wu Z, Xu X, Xiong W, Wu P, Chen Y, Li M, et al Genome-Wide Analysis of the NAC Gene Family in Physic Nut (*Jatropha curcas L*.). PloS ONE. 2015;10(6):e0131890 10.1371/journal.pone.0131890 26125188 PMC4488383

[22] Ma J, Wang F, Li M-Y, Jiang Q, Tan G-F, Xiong A-S. Genome wide analysis of the NAC transcription factor family in Chinese cabbage to elucidate responses to temperature stress. Scientia Horticulturae. 2014;165:82–90. 10.1016/j.scienta.2013.11.005

[23] Souer E, Van H A, Kloos D, et al The no apical meristem gene of Petunia is required for pattern formation in embryos and flowers and is expressed at meristem and primordia boundaries. Cell. 1996;85(2):159–70. 8612269 10.1016/s0092-8674(00)81093-4

[24] Duval M, Hsieh T F, Kim S Y, et al Molecular characterization of AtNAM: a member of the Arabidopsis NAC domain superfamily, Plant Molecular Biology. 2002;50(2):237–48. 12175016 10.1023/a:1016028530943

[25] He XJ, Mu RL, Cao WH, Zhang ZG, Zhang JS, Chen SY. AtNAC2, a transcription factor downstream of ethylene and auxin signaling pathways, is involved in salt stress response and lateral root development. The Plant Journal. 2005;44(6):903–16. 10.1111/j.1365-313X.2005.02575.x 16359384

[26] Hao YJ, Wei W, Song QX, Chen HW, Zhang YQ, Wang F, et al Soybean NAC transcription factors promote abiotic stress tolerance and lateral root formation in transgenic plants. The Plant Journal. 2011;68(2):302–13. 10.1111/j.1365-313X.2011.04687.x 21707801

[27] Zhong R, Lee C, Ye ZH. Functional characterization of poplar wood-associated NAC domain transcription factors. Plant Physiology. 2010;152(2):1044–55. 10.1104/pp.109.148270 19965968 PMC2815876

[28] Guo Y, Gan S. AtNAP, a NAC family transcription factor, has an important role in leaf senescence. The Plant Journal. 2006;46(4):601–12. 10.1111/j.1365-313X.2006.02723.x 16640597

[29] Kjaersgaard T, Jensen MK, Christiansen MW, Gregersen P, Kragelund BB, Skriver K. Senescence-associated barley NAC (NAM, ATAF1,2, CUC) transcription factor interacts with radical-induced cell death 1 through a disordered regulatory domain. The Journal of Biological Chemistry. 2011;286(41):35418–29. 10.1074/jbc.M111.247221 21856750 PMC3195629

[30] Yang SD, Seo PJ, Yoon HK, Park CM. The Arabidopsis NAC transcription factor VNI2 integrates abscisic acid signals into leaf senescence via the COR/RD genes. The Plant Cell. 2011;23(6):2155–68. 10.1105/tpc.111.084913 21673078 PMC3160032

[31] Sperotto RA, Ricachenevsky FK, Duarte GL, Boff T, Lopes KL, Sperb ER, et al Identification of up-regulated genes in flag leaves during rice grain filling and characterization of OsNAC5, a new ABA-dependent transcription factor. Planta. 2009;230(5):985–1002. 10.1007/s00425-009-1000-9 19697058

[32] Morishita T, Kojima Y, Maruta T, et al Arabidopsis NAC transcription factor, ANAC078, regulates flavonoid biosynthesis under high-light. Plant & Cell Physiology. 2014; 50(12):2210–2222. 10.1093/pcp/pcp159 19887540

[33] Tran LS, Nakashima K, Sakuma Y, Simpson SD, Fujita Y, Maruyama K, et al Isolation and functional analysis of Arabidopsis stress-inducible NAC transcription factors that bind to a drought-responsive cis-element in the early responsive to dehydration stress 1 promoter. The Plant Cell. 2004;16(9):2481–98. 10.1105/tpc.104.022699 15319476 PMC520947

[34] Nakashima K, Takasaki H, Mizoi J, Shinozaki K, Yamaguchi-Shinozaki K. NAC transcription factors in plant abiotic stress responses. Biochimica et Biophysica Acta. 2012;1819(2):97–103. 10.1016/j.bbagrm.2011.10.005 22037288

[35] Wu Y, Deng Z, Lai J, Zhang Y, Yang C, Yin B, et al Dual function of Arabidopsis ATAF1 in abiotic and biotic stress responses. Cell research. 2009;19(11):1279–90. 10.1038/cr.2009.108 19752887

[36] Nakashima K, Tran LS, Van Nguyen D, Fujita M, Maruyama K, Todaka D, et al Functional analysis of a NAC-type transcription factor OsNAC6 involved in abiotic and biotic stress-responsive gene expression in rice. The Plant Journal. 2007;51(4):617–30. 10.1111/j.1365-313X.2007.03168.x 17587305

[37] Hu H, You J, Fang Y, Zhu X, Qi Z, Xiong L. Characterization of transcription factor gene SNAC2 conferring cold and salt tolerance in rice. Plant Molecular Biology. 2008;67(1–2):169–81. 10.1007/s11103-008-9309-5 18273684

[38] Jeong JS, Kim YS, Redillas MC, Jang G, Jung H, Bang SW, et al OsNAC5 overexpression enlarges root diameter in rice plants leading to enhanced drought tolerance and increased grain yield in the field. Plant Biotechnology Journal. 2013;11(1):101–14. 10.1111/pbi.12011 23094910

[39] Wu Z J, Li X H, Liu Z W, et al *De novo* assembly and transcriptome characterization: Novel insights into catechins biosynthesis in *Camellia sinensis*[J]. BMC Plant Biology, 2014, 14(1):1–16. 25316555 10.1186/s12870-014-0277-4 PMC4203915

[40] Wu ZJ, Li XH, Liu ZW, Li H, Wang YX, Zhuang J. Transcriptome-based discovery of AP2/ERF transcription factors related to temperature stress in tea plant (*Camellia sinensis*). Functional & Integrative Genomics. 2015;15(6):741–52. 10.1007/s10142-015-0457-9 26233577

[41] Wu ZJ, Li XH, Liu ZW, Li H, Wang YX, Zhuang J. Transcriptome-wide identification of Camellia sinensis WRKY transcription factors in response to temperature stress. Molecular Genetics And Genomics. 2015 10.1007/s00438-015-1107-6 26308611

[42] Liu ZW, Wu ZJ, Li XH, Huang Y, Li H, Wang YX, et al Identification, classification, and expression profiles of heat shock transcription factors in tea plant (Camellia sinensis) under temperature stress. Gene. 2016;576(1 Pt 1):52–9. 10.1016/j.gene.2015.09.076 26431998

[43] Cao H, Wang L, Yue C, Hao X, Wang X, Yang Y. Isolation and expression analysis of 18 CsbZIP genes implicated in abiotic stress responses in the tea plant (Camellia sinensis). Plant Physiology and Biochemistry. 2015;97:432–42. 10.1016/j.plaphy.2015.10.030 26555901

[44] Tamura K, Peterson D, Peterson N, Stecher G, Nei M, Kumar S. MEGA5: molecular evolutionary genetics analysis using maximum likelihood, evolutionary distance, and maximum parsimony methods. Molecular Biology and Evolution. 2011;28(10):2731–9. 10.1093/molbev/msr121 21546353 PMC3203626

[45] Wu ZJ, Tian C, Jiang Q, Li XH, Zhuang J. Selection of suitable reference genes for qRT-PCR normalization during leaf development and hormonal stimuli in tea plant (*Camellia sinensis*). Scientific Reports. 2016;6:19748 10.1038/srep19748 26813576 PMC4728435

[46] Pfaffl M W. A new mathematical model for relative quantification in real-time RT-PCR. Nucleic Acids Research. 2001; 29(9): e45 11328886 10.1093/nar/29.9.e45 PMC55695

[47] Elofsson JH A. Hidden Markov models that use predicted secondary structures for fold recognition. 1999. 10373007

[48] Elbling L, Weiss RM, Teufelhofer O, Uhl M, Knasmueller S, Schulte-Hermann R, et al Green tea extract and (-)-epigallocatechin-3-gallate, the major tea catechin, exert oxidant but lack antioxidant activities. FASEB journal: official publication of the Federation of American Societies for Experimental Biology. 2005;19(7):807–809. 10.1096/fj.04-2915fje 15738004

[49] Fang Y, You J, Xie K, Xie W, Xiong L. Systematic sequence analysis and identification of tissue-specific or stress-responsive genes of NAC transcription factor family in rice. Molecular Genetics and Genomics. 2008;280(6):547–563. 10.1007/s00438-008-0386-6 18813954

[50] Christianson J A, Dennis E S, Llewellyn D J, et al ATAF NAC transcription factors: Regulators of plant stress signaling. Plant Signaling & Behavior. 2010; 5(4):428–432. 20118664 10.4161/psb.5.4.10847 PMC7080423

[51] Huang H, Wang Y, Wang S, Wu X, Yang K, Niu Y, et al Transcriptome-wide survey and expression analysis of stress-responsive NAC genes in *Chrysanthemum lavandulifolium*. Plant Science. 2012;193–194:18–27. 10.1016/j.plantsci.2012.05.004 22794915

[52] Xu ZY, Kim SY, Hyeon do Y, Kim DH, Dong T, Park Y, et al The Arabidopsis NAC transcription factor ANAC096 cooperates with bZIP-type transcription factors in dehydration and osmotic stress responses. The Plant Cell. 2013;25(11):4708–24. 10.1105/tpc.113.119099 24285786 PMC3875745

[53] Shang H, Li W, Zou C, Yuan Y. Analyses of the NAC transcription factor gene family ingossypium raimondiiulbr.: chromosomal location, structure, phylogeny, and expression patterns. Journal of Integrative Plant Biology. 2013;55(7):663–76. 10.1111/jipb.12085 23756542

[54] Greve K, La Cour T, Jensen MK, Poulsen FM, Skriver K. Interactions between plant RING-H2 and plant-specific NAC (NAM/ATAF1/2/CUC2) proteins: RING-H2 molecular specificity and cellular localization. The Biochemical Journal. 2003;371(Pt 1):97–108. 10.1042/BJ20021123 12646039 PMC1223272

[55] Singh AK, Sharma V, Pal AK, Acharya V, Ahuja PS. Genome-wide organization and expression profiling of the NAC transcription factor family in potato (*Solanum tuberosum* L.). DNA Research. 2013;20(4):403–23. 10.1093/dnares/dst019 23649897 PMC3738166

[56] Kim S-G, Lee S, Ryu J, Park C-M. Probing protein structural requirements for activation of membrane-bound NAC transcription factors in Arabidopsis and rice. Plant Science. 2010;178(3):239–44. 10.1016/j.plantsci.2009.12.007

[57] Seo PJ, Kim SG, Park CM. Membrane-bound transcription factors in plants. Trends in plant science. 2008;13(10):550–6. 10.1016/j.tplants.2008.06.008 18722803

[58] Kim SG, Lee S, Seo PJ, Kim SK, Kim JK, Park CM. Genome-scale screening and molecular characterization of membrane-bound transcription factors in Arabidopsis and rice. Genomics. 2010;95(1):56–65. 10.1016/j.ygeno.2009.09.003 19766710

[59] Lee S, Lee HJ, Huh SU, Paek KH, Ha JH, Park CM. The Arabidopsis NAC transcription factor NTL4 participates in a positive feedback loop that induces programmed cell death under heat stress conditions. Plant Science. 2014;227:76–83. 10.1016/j.plantsci.2014.07.003 25219309

[60] Zhang H, Huang Y, Zhang H, Huang Y. Genome-wide survey and characterization of greenbug induced nac transcription factors in sorghum [*Sorghum bicolor* (L.) Moench]. Plant & Animal Genome. 2013.

[61] Liu T, Song X, Duan W, Huang Z, Liu G, Li Y, et al Genome-wide analysis and expression patterns of nac transcription factor family under different developmental stages and abiotic stresses in Chinese cabbage. Plant Molecular Biology Reporter. 2014;32(5):1041–56. 10.1007/s11105-014-0712-6

[62] Ha C V, Esfahani M N, Watanabe Y, et al Genome-wide identification and expression analysis of the CaNAC family members in chickpea during development, dehydration and ABA treatments.[J]. Plos ONE, 2014, 9(12):e114107–e114107. 10.1371/journal.pone.0114107 25479253 PMC4257607

[63] You J, Zhang L, Song B, Qi X, Chan Z. Systematic analysis and identification of stress-responsive genes of the NAC gene family in Brachypodium distachyon. PloS ONE. 2015;10(3):e0122027 10.1371/journal.pone.0122027 25815771 PMC4376915

[64] Wang X, Wang H, Wang J, Sun R, Wu J, Liu S, et al The genome of the mesopolyploid crop species Brassica rapa. Nature Genetics. 2011;43(10):1035–9. 10.1038/ng.919 21873998

[65] Li N, Yang Y, Ye J, Lu J, Zheng X, Liang Y. Effects of sunlight on gene expression and chemical composition of light-sensitive albino tea plant. Plant Growth Regulation. 2015;78(2):253–62. 10.1007/s10725-015-0090-6

[66] Le DT, Nishiyama R, Watanabe Y, Mochida K, Yamaguchi-Shinozaki K, Shinozaki K, et al Genome-wide survey and expression analysis of the plant-specific NAC transcription factor family in soybean during development and dehydration stress. DNA Research. 2011;18(4):263–76. 10.1093/dnares/dsr015 21685489 PMC3158466

[67] Xie Qi F G, Colgan Diana and Chua Nam-Hai. Arabidopsis NAC1 transduces auxin signal downstream of TIR1 to promote lateral root development. Genome Research. 2000;12(1):47–56. 10.1101/gad.852200 PMC317103 11114891

[68] Kato H, Motomura T, Komeda Y, Saito T, Kato A. Overexpression of the NAC transcription factor family gene ANAC036 results in a dwarf phenotype in Arabidopsis thaliana. J Plant Physiol. 2010;167(7):571–7. 10.1016/j.jplph.2009.11.004 19962211

[69] Sanghera G S, Wani S H, Hussain W, et al Engineering cold stress tolerance in crop plants.[J]. Current Genomics. 2011; 12(1):30–43. 10.2174/138920211794520178 21886453 PMC3129041

[70] Olsen AN, Ernst HA, Leggio LL, Skriver K. NAC transcription factors: structurally distinct, functionally diverse. Trends in Plant Science. 2005;10(2):79–87. 10.1016/j.tplants.2004.12.010 15708345

[71] Zeller G, Henz SR, Widmer CK, Sachsenberg T, Ratsch G, Weigel D, et al Stress-induced changes in the Arabidopsis thaliana transcriptome analyzed using whole-genome tiling arrays. The Plant Journal. 2009;58(6):1068–82. 10.1111/j.1365-313X.2009.03835.x 19222804

[72] Mittler R, Blumwald E. The roles of ROS and ABA in systemic acquired acclimation. The Plant Cell. 2015;27(1):64–70. 10.1105/tpc.114.133090 25604442 PMC4330577

[73] Fujita M, Fujita Y, Maruyama K, Seki M, Hiratsu K, Ohme-Takagi M, et al A dehydration-induced NAC protein, RD26, is involved in a novel ABA-dependent stress-signaling pathway. The Plant Journal. 2004;39(6):863–76. 10.1111/j.1365-313X.2004.02171.x. 15341629

