# Supplementary material for: Transcriptome-Wide Identification and Expression Analysis of the NAC Gene Family in Tea Plant [Camellia sinensis (L.) O. Kuntze]
Source: PLoS One. 2016 Nov 17;11(11):e0166727. doi: 10.1371/journal.pone.0166727 (PMC5113971; doi:10.1371/journal.pone.0166727)
Supplement: S1 Fig — The gene sequence from Tea_T1 was determined by RNA-Seq; the gene sequence from ‘Yingshuang’ was determined by PCR sequencing. (DOC) [file pone.0166727.s001.doc]

**S1 Fig. Comparison of *CsNAC16* gene sequences determined by RNA-Seq and PCR sequencing.**

The gene sequence from Tea_T1 was determined by RNA-Seq; the gene sequence from ‘Yingshuang’ was determined by PCR sequencing.
