# Supplementary figures and images for: Transcriptome-Wide Identification and Expression Analysis of the NAC Gene Family in Tea Plant [Camellia sinensis (L.) O. Kuntze]
Source: PLoS One. 2016 Nov 17;11(11):e0166727. doi: 10.1371/journal.pone.0166727 (PMC5113971; doi:10.1371/journal.pone.0166727)

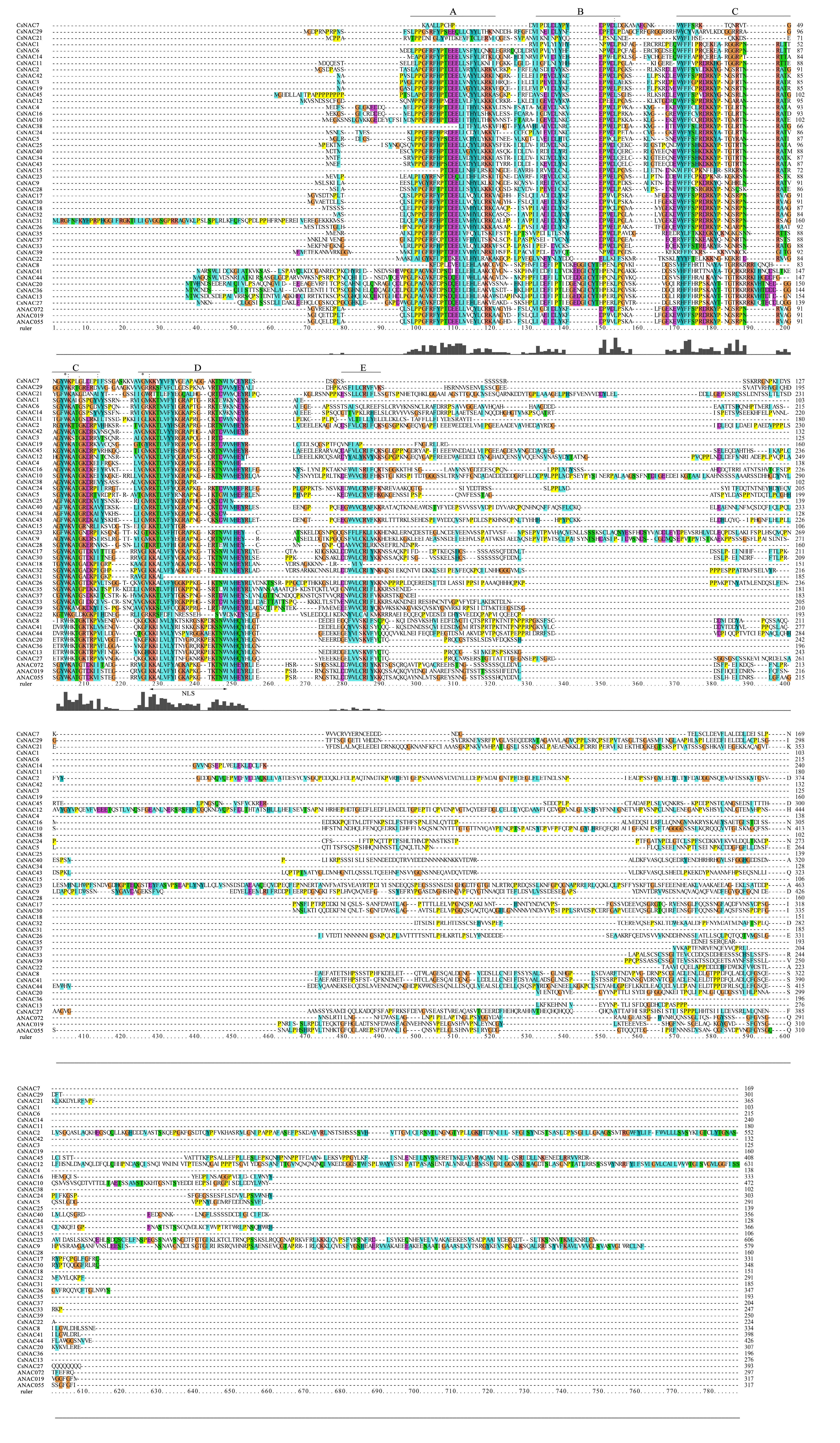

Supplement: S9 Fig — Conserved NAC domain is divided into five subdomains (A–E), which are indicated by lines above the sequences. The putative nuclear localization signal (NLS) is shown by a double-headed arrow below the sequences. (PNG) [file pone.0166727.s009.png]

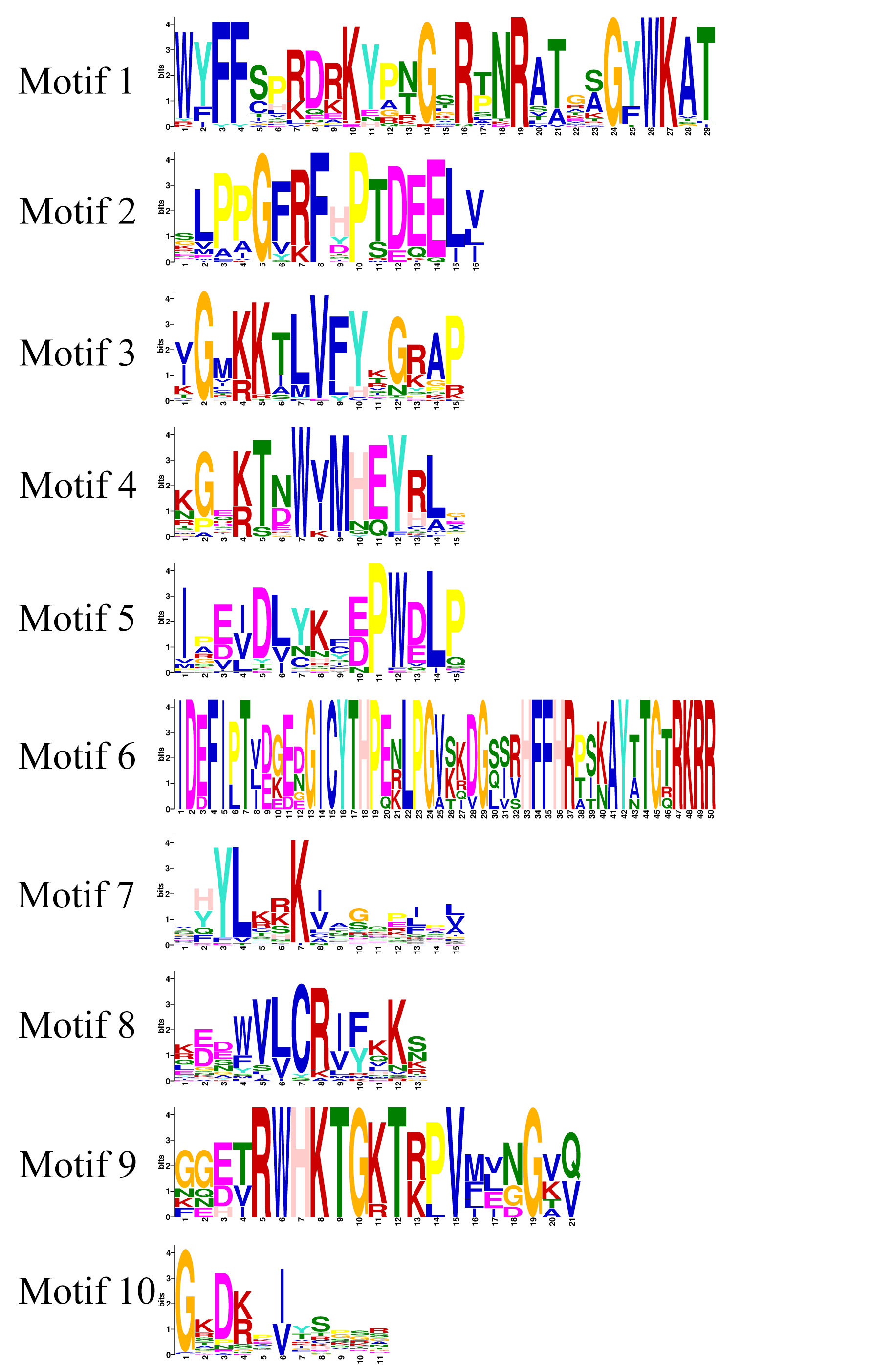

Supplement: S10 Fig — (PNG) [file pone.0166727.s010.png]
